# Supplementary material for: Novel Driver Strength Index highlights important cancer genes in TCGA PanCanAtlas patients
Source: PeerJ. 2022 Aug 11;10:e13860. doi: 10.7717/peerj.13860 (PMC9375969; doi:10.7717/peerj.13860)
Supplement: Supplemental Information 2 [file peerj-10-13860-s002.zip › COHORTS/genes plots/cohorts/2021_8_16_14_9_distribution_cohorts_CESC.pdf]

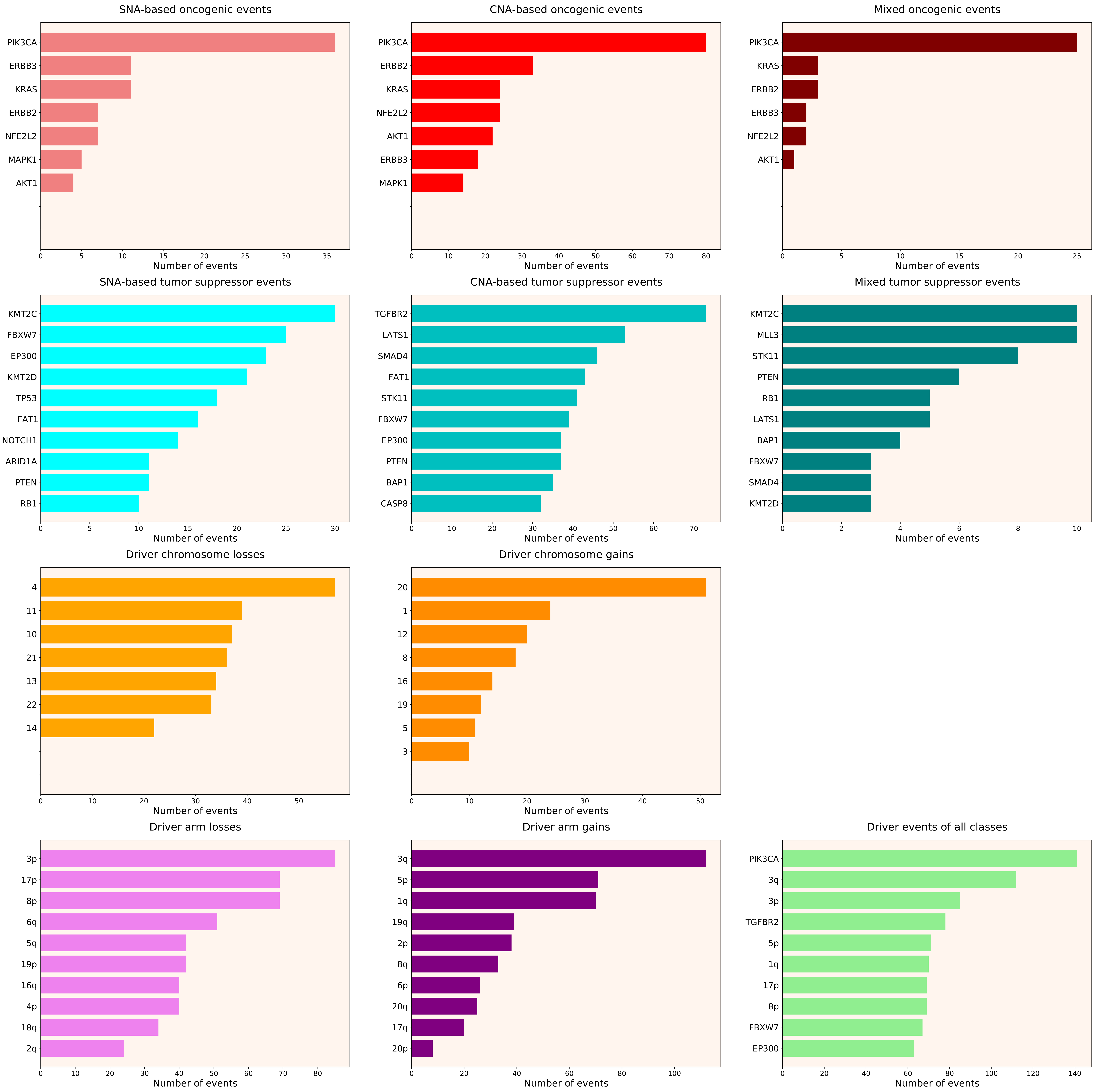

SNA-based oncogenic events
PIK3CA
ERBB3
KRAS
ERBB2
NFE2L2
MAPK1
AKT1
0 5 10 15 20 25 30 35
Number of events
CNA-based oncogenic events
PIK3CA
ERBB2
KRAS
NFE2L2
AKT1
ERBB3
MAPK1
0 10 20 30 40 50 60 70 80
Number of events
Mixed oncogenic events
PIK3CA
KRAS
ERBB2
ERBB3
NFE2L2
AKT1
0 5 10 15 20 25
Number of events
SNA-based tumor suppressor events
KMT2C
FBXW7
EP300
KMT2D
TP53
FAT1
NOTCH1
ARID1A
PTEN
RB1
0 5 10 15 20 25 30
Number of events
CNA-based tumor suppressor events
TGFBR2
LATS1
SMAD4
FAT1
STK11
FBXW7
EP300
PTEN
BAP1
CASP8
0 10 20 30 40 50 60 70
Number of events
Mixed tumor suppressor events
KMT2C
MLL3
STK11
PTEN
RB1
LATS1
BAP1
FBXW7
SMAD4
KMT2D
0 2 4 6 8 10
Number of events
Driver chromosome losses
4
11
10
21
13
22
14
0 10 20 30 40 50
Number of events
Driver chromosome gains
20
1
12
8
16
19
5
3
0 10 20 30 40 50
Number of events
Driver arm losses
3p
17p
8p
6q
5q
19p
16q
4p
18q
2q
0 10 20 30 40 50 60 70 80
Number of events
Driver arm gains
3q
5p
1q
19q
2p
8q
6p
20q
17q
20p
0 20 40 60 80 100
Number of events
Driver events of all classes
PIK3CA
3q
3p
TGFBR2
5p
1q
17p
8p
FBXW7
EP300
0 20 40 60 80 100 120 140
Number of events
